# Blocking Extracellular Cyclophilin A‐Mediated Adhesion Protects Against Bacterial Infection

**DOI:** 10.1002/mco2.71063

**Published:** 2026-10-13

**Authors:** Xiaoyuan Bai, Heqiao Li, Pengtao Jiao, Yuna Zhao, Xiaoxiao Jia, Han Zhong Pei, Zeqiu Fan, Runshan Lin, He Zhang, Beinan Wang, Xinhai Chen, Yuhai Bi, De Chang, Zhongfa Zhang, Yuxin Chen, Wenjun Liu, Lei Sun

**Affiliations:** ^1^ Key Laboratory of Pathogenic Microbiology and Immunology Institute of Microbiology Chinese Academy of Sciences Beijing China; ^2^ Beijing Institute of Heart Lung and Blood Vessel Diseases Beijing Anzhen Hospital Capital Medical University Beijing China; ^3^ Institute of Infectious Diseases Shenzhen Bay Laboratory Shenzhen China; ^4^ Institute of Animal Science Chinese Academy of Agricultural Sciences Beijing China; ^5^ National Institute of Pathogen Biology Chinese Academy of Medical Sciences & Peking Union Medical College Beijing China; ^6^ Shenzhen Medical Academy of Research and Translation (SMART) Shenzhen China; ^7^ Medical School University of Chinese Academy of Sciences Beijing China; ^8^ Department of Pulmonary and Critical Care Medicine at The Seventh Medical Center College of Pulmonary and Critical Care Medicine of The Eighth Medical Center Chinese PLA General Hospital Beijing China; ^9^ Public Health Clinical Center Affiliated to Shandong University Shandong Public Health Clinical Center Jinan China; ^10^ Department of Laboratory Medicine Nanjing Drum Tower Hospital, The Affiliated Hospital of Medical School Nanjing University Nanjing China

**Keywords:** adhesion receptor, bacterial infection, cyclophilin A, host‐directed therapy, inflammatory response, TGF‐β

## Abstract

Bacterial pneumonia remains a leading cause of mortality and morbidity worldwide and is primarily managed through antibiotic therapy. However, the increasing threat of antimicrobial resistance underscores the urgent need for alternative strategies. In this study, we identify extracellular cyclophilin A (eCypA) as a key host factor that promotes the adherence of bacteria such as group A *Streptococcus* (GAS) and *Streptococcus pneumoniae* (*S. P*.). Using single‐nucleus RNA sequencing of mouse lung tissue, we demonstrate that eCypA upregulates the expression of integrin/extracellular matrix adhesion receptors, including integrin α5, fibronectin, and mucin, an effect largely mediated by enhancing the transforming growth factor‐β (TGF‐β) signaling. Mechanistically, eCypA interacts with TGF‐β receptor II, acting as a coactivator to increase its binding to TGF‐β1 and amplify downstream signaling. Notably, administration of an anti‐CypA monoclonal antibody yields both prophylactic and therapeutic effects against GAS‐induced lung damage in mice by reducing bacterial adhesion and suppressing proinflammatory cytokine production. Collectively, these findings identify eCypA as a critical regulator of infection exploited by diverse bacteria that rely on integrin/extracellular matrix for adherence, highlighting eCypA as a promising target for host‐directed therapies designed to mitigate bacterial attachment and subsequent inflammation.

## Graphical abstract

eCypA promotes bacterial infection by upregulating TGF‐β signaling‐dependent adhesion receptor expression. Pathogens such as IAV, GAS, *S. P*., and *K. P*. induce eCypA secretion. Binding of eCypA with TβR2 enhances the affinity of TGF‐β1 and TβR2, which positively regulates adhesion receptors expression via the TGF‐β/Smad signaling pathway, thereby facilitating bacterial adhesion. These functions of eCypA were suppressed by anti‐CypA mAb.

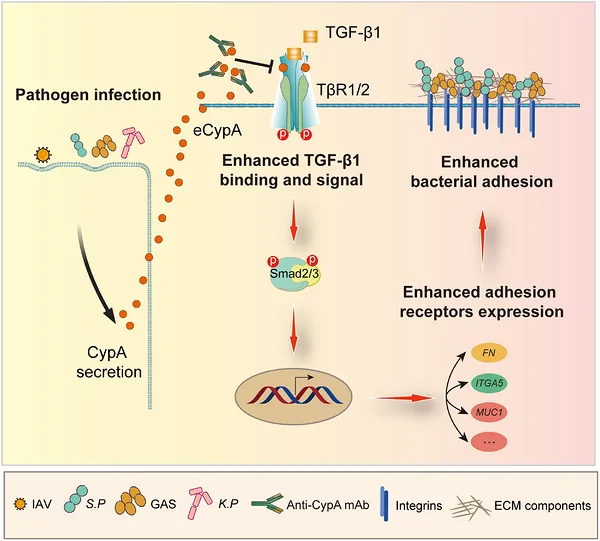

## 1 Introduction

Bacterial pneumonia and influenza A virus (IAV)‐induced secondary bacterial pneumonia occur frequently and are prominent causes of death in clinical settings, particularly in children and older patients [1, 2, 3]. Although antibiotic therapy is the primary approach used to control bacterial infections, antimicrobial resistance has become a major threat to public health worldwide [4, 5]. By 2050, antibiotic‐resistant bacterial infections may cause 39 million deaths and result in annual economic losses of up to $412 billion [6]. Host‐directed therapy (HDT) is a promising approach for overcoming antimicrobial resistance mainly by targeting host immune mechanisms, such as by enhancing the ability of immune cells to ingest or clear bacteria [7, 8, 9, 10]. Identifying key host proteins that regulate bacterial infection may provide additional strategies for HDT.

For many bacteria, adherence to host cells and tissues is a critical initial step in establishing infection and is dependent on the binding of bacterial membrane proteins to their receptors on the host cell surface [11, 12]. The extent to which specific adhesion receptors, such as integrins, extracellular matrix (ECM) components, CD46, platelet‐activating factor receptor (PAFR), mucins, and mannose‐containing glycans, are utilized varies across bacterial species [13, 14, 15, 16, 17, 18, 19, 20]. For example, group A *Streptococcus* (GAS) and *S. pneumoniae* (*S. P*.) predominantly exploit the integrin/ECM axis, using surface adhesins that bind fibronectin or plasminogen to promote integrin engagement [13, 14, 17]. In contrast, *Klebsiella pneumoniae* (*K. P*.) relies primarily on type 1 fimbriae–mediated, mannose‐sensitive adhesion, in which FimH directly recognizes terminal D‐mannose residues on host glycoproteins [13, 20]. Together, these mechanistically distinct strategies suggest that a host factor that modulates the integrin/ECM pathway could differentially influence bacteria that depend on integrin/ECM‐dependent versus mannose‐dependent adhesion routes. Upregulation of bacterial adhesion receptor expression is a major driver of IAV‐enhanced host susceptibility to bacterial infections, including *S. P*., GAS, *Staphylococcus aureus* (*S. A*.), and *Haemophilus influenza* infection, which require integrins and ECM as adherence receptors [13, 21, 22, 23]. Therefore, targeting bacterial adhesion receptors or key regulators of bacterial adhesion receptor expression is a promising HDT for controlling bacterial infection or IAV‐induced bacterial coinfection.

Cyclophilin A (CypA, encoded by *PPIA*) is a peptidyl‐prolyl *cis/trans* isomerase (PPIase) [24] that is an important regulator of infection and inflammation [25]. CypA is distributed mainly in the cytoplasm of resting cells and is released into the extracellular space in response to oxidative stress, injury, and hypoxia [26, 27, 28]. Intracellular CypA (iCypA) and extracellular cyclophilin A (eCypA) regulate infection and inflammation through different mechanisms. iCypA promotes inflammatory responses by regulating the ubiquitination of nuclear factor κB P65 subunit [29], acid‐inducible gene I, mitochondrial antiviral‐signaling protein [30], gp130 [31], and interleukin‐1β [32]. In addition, eCypA binds to the cell surface via its receptors and activates multiple intracellular signaling pathways, leading to proinflammatory cytokine production, leukocyte migration, endothelial cell (EC) activation, keratinocyte proliferation, and Th17 differentiation [28, 33, 34, 35, 36, 37, 38, 39, 40]. We previously demonstrated that iCypA upregulated the IAV‐activated FAK/Akt signaling pathway and subsequently increased integrin α5 expression and actin rearrangement to facilitate GAS coinfection [23]. Furthermore, eCypA is upregulated in response to respiratory viral infection, which promotes virus‐induced inflammatory responses by enhancing leukocyte migration [37]. However, the role of eCypA in bacterial infection remains unclear.

In this study, we investigated the role of eCypA in bacterial respiratory infections. We found that eCypA, which acts as a coactivator of the transforming growth factor β (TGF‐β) signaling pathway, promoted the expression of bacterial adhesion receptors. The monoclonal antibody against CypA (anti‐CypA mAb) reduced bacterial adherence and alleviated bacterial pneumonia, suggesting that eCypA is a target for treating bacterial pneumonia.

## 2 Results

### 2.1 eCypA Is Critical for the Adhesion of GAS and *S. P*. 

Our previous studies demonstrated that both iCypA and eCypA are upregulated during IAV infection [23, 37] and that iCypA promotes integrin α5 expression and actin rearrangement to facilitate GAS coinfection. In this study, we further investigated the contribution of eCypA to IAV‐induced bacterial coinfections. Mice infected with IAV were subsequently challenged with either GAS or *S. P*. and treated with anti‐CypA mAb or control immunoglobulin G (IgG) (Figure 1A). Bacterial adherence assays revealed that the numbers of colony‐forming units (CFUs) of GAS (Figure 1B) and *S. P*. (Figure 1C) in the lungs of anti‐CypA mAb‐treated mice were much lower than those in the lungs of IgG‐treated mice. Moreover, we investigated the effect of anti‐CypA mAb on the pathogenicity of IAV‐GAS coinfection. In line with the observed decrease in bacterial adhesion, mice treated with anti‐CypA mAb exhibited lower lung indices (Figure 1D) and slower weight loss (Figure 1E). Similarly, anti‐CypA mAb treatment significantly alleviated lung consolidation, with less infiltration of leukocytes, hemorrhage, and edema (Figure 1F). We also tested the effect of anti‐CypA mAb on the expression of inflammatory cytokines. The results of quantitative polymerase chain reaction (qPCR) revealed that the anti‐CypA mAb suppressed the expression of proinflammatory cytokines, such as *Tnfa*, *Il1b*, *Il6*, *Ccl2*, and *Cxcl10* in the lungs of IAV‐GAS‐coinfected mice (Figure 1G). Moreover, the anti‐CypA mAb suppressed GAS (Figure 1H) and *S. P*. (Figure 1I) adherence in IAV‐infected A549 cells (type II alveolar epithelial cell line). These results demonstrate that targeting eCypA inhibits IAV‐induced GAS and *S. P*. adherence and mouse pneumonia, suggesting that eCypA is critical for bacterial adherence.

**FIGURE 1:**
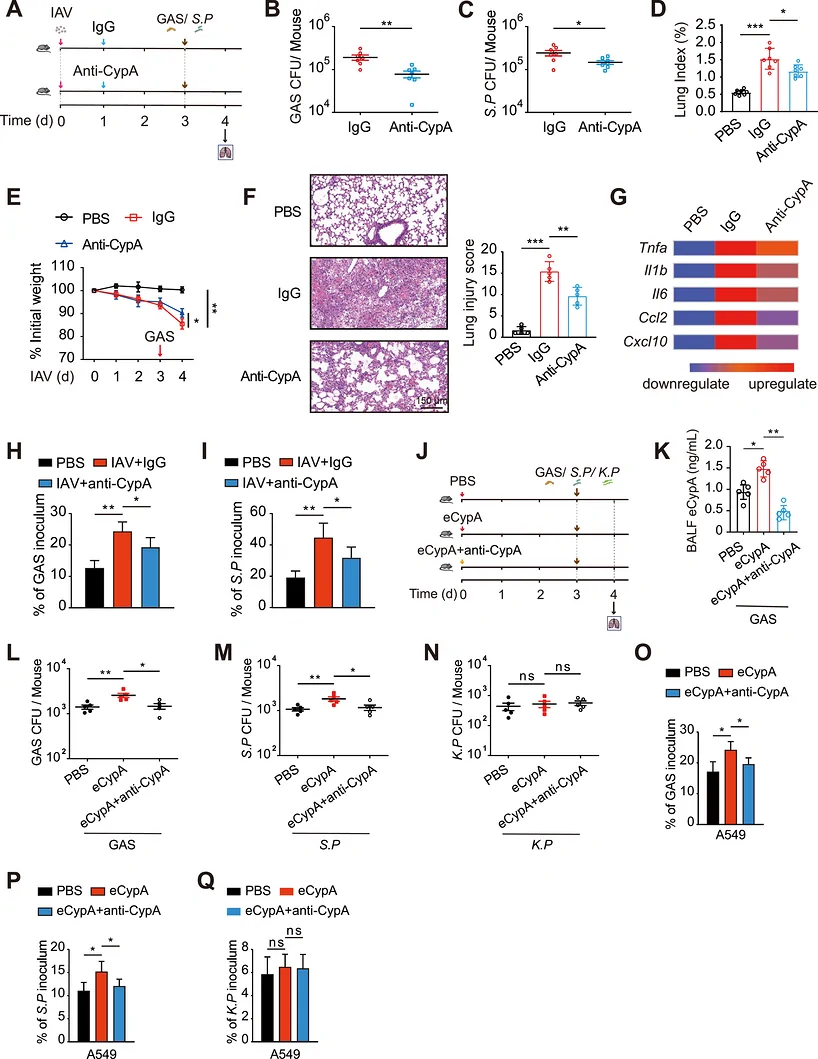
eCypA is critical for the adhesion of GAS and *S.P* (A) C57BL/6 mice (*n* = 7) were infected intranasally with PBS or 3000 PFU of influenza A/WSN/33 (H1N1) on Day 0 followed by intraperitoneal injection of isotype IgG (IgG) or anti‐CypA mAb (100 µg/mouse) on Day 1, and then infected intranasally with GAS (1.0 × 10^7^ CFU), *S. P* (1.0 × 10^7^ CFU), *K. P* (1.0 × 10^7^ CFU), or PBS on Day 3. (B) Adherence of GAS in the lungs of C57BL/6 mice on Day 4. (C) Adherence of *S. P* in the lungs of C57BL/6 mice on Day 4. (D) The lung index (100× lung/body weight) was calculated on Day 4. (E) The body weights of C57BL/6 mice were monitored during Days 0–4. (F) Hematoxylin and eosin staining of mouse lungs on Day 4 (left). The severity of lung injury was analyzed in a blinded manner (right). Scale bar, 150 µm. (G) Heatmap showing the results of the qPCR analysis indicating the mRNA levels of proinflammatory cytokines in the lungs of C57BL/6 mice on Day 4. (H and I) Adherence of GAS (H) and *S. P* (I) in A549 cells infected with PBS or IAV (MOI = 1) followed by treatment with IgG or anti‐CypA mAb (50 nM) for 24 h and then infection of the cells with GAS (MOI = 10) for 2 h. Data are reported as the percentage of adherent bacteria relative to the input inoculum. (J) C57BL/6 mice were preinjected intraperitoneally with eCypA (40 µg/mouse) or vehicle (PBS) for 2 h, followed by intraperitoneal injection with anti‐CypA mAb (100 µg/mouse) or vehicle (PBS) on Day 0 and then infected intranasally with GAS (1.0 × 10^7^ CFU), *S. P* (1.0 × 10^7^ CFU), *K. P* (1.0 × 10^7^ CFU), or PBS on Day 3. (K) ELISA of eCypA in BALF from mice infected with GAS. (L–N) Adherence of GAS (K), *S. P* (L), and *K. P* (M) in the lungs of C57BL/6 mice (*n* = 5) on Day 4. (O–Q) Adherence assays of GAS (N), *S. P* (O), and *K. P* (P) in A549 cells pretreated with PBS, eCypA (10 nM) or eCypA + anti‐CypA mAb (10 nM eCypA + 50 nM anti‐CypA mAb, preincubated at 37°C for 1 h) for 12 h, followed by GAS, *S. P*, or *K. P* (MOI = 10) infection for 2 h. Data are shown as the percentage of adherent bacteria relative to the input inoculum. Data shown in (B–F) and (K–N) are presented as the means ± SEMs; Data shown in (H), (I), and (O–Q) are presented as the means ± SDs; *p < 0.05, **p < 0.01, ***p < 0.001 and ns = not significant. Unpaired two‐tailed Student's *t* test for (B) and (C), and one‐way ANOVA followed by Dunnett's test for (D–F), (H), (I), and (K–Q).

Next, three representative pneumonia‐causing bacteria, GAS, *S. P*., and *K. P*., were selected to further investigate the effect of eCypA on bacterial adherence. GAS and *S. P*. require integrin/ECM components as adherence receptors [41, 42, 43], whereas *K. P*. relies mainly on mannose to adhere to its host [13, 20]. Mice were treated with eCypA, eCypA + anti‐CypA mAb, or PBS control and then infected with GAS, *S. P*., or *K. P*. (Figure 1J). Before measuring adherence, we confirmed that bacterial infection alone triggered eCypA release. Single GAS inoculation elevated eCypA levels in the bronchoalveolar lavage fluid (BALF) of both PBS‐treated and exogenous eCypA‐treated mice, and this increase was reversed by anti‐CypA mAb (Figure 1K). To determine whether this is a general response, we extended our analysis to clinical samples, animal models, and cell cultures. Serum from outpatients with GAS or *S. P*. infection had significantly higher eCypA levels than serum from healthy donors did (Figure S1A). In parallel, mouse models infected with GAS, *S. P*., or *K. P*. (Figure S1B) showed a rapid increase in eCypA in the BALF and serum at 1 day post‐infection (d.p.i.) and maintained high levels up to 5 d.p.i (Figure S1C–H). Consistently, high levels of eCypA were observed in the supernatants of GAS‐, *S. P*.‐, or *K. P.‐* infected A549 cells and primary human tonsil fibroblasts (PHTF) at 24 h post infection (Figure S1I). Together, these data demonstrate that bacterial respiratory infection induces eCypA secretion.

We next investigated the effect of eCypA on bacterial adhesion in GAS‐, *S. P*.‐, or *K. P*.‐ infected mice. Treatment with eCypA increased the number of GAS (Figure 1L) and *S. P*. (Figure 1M) that adhered to the lungs of mice and anti‐CypA mAb attenuated the effect of eCypA. However, eCypA did not affect *K. P*. adherence in mice (Figure 1N). Furthermore, the effect of eCypA on bacterial adherence was also evaluated in cell models. The numbers of GAS (Figure 1O and Figure S2A) and *S. P*. (Figure 1P and Figure S2B) were greater in eCypA‐treated A549 cells and PHTF than in PBS‐treated cells, and were reduced by the anti‐CypA mAb. However, eCypA did not affect the *K. P*. adherence of A549 cells or PHTF (Figure 1Q and Figure S2C). Notably, in CypA‐knockdown A549 cells (A549/CypA‐), eCypA still promoted GAS and *S. P*. adherence (Figure S2D,E), indicating that eCypA acts independently of iCypA. Collectively, these results demonstrate that eCypA promotes the adherence of GAS and *S. P*., but not *K. P*., suggesting that eCypA may selectively enhance integrin/ECM‐dependent bacterial adherence.

### 2.2 eCypA Upregulates the Expression of Bacterial Adhesion Receptors

To elucidate the mechanism by which eCypA promotes bacterial adherence, we performed single‐nucleus RNA sequencing (snRNA‐seq) of lungs from GAS‐infected mice, as shown in Figure 1J. A total of 18,835 single nuclei were sequenced, with 6066 from PBS‐treated mice, 7405 from eCypA‐treated mice, and 5364 from eCypA + anti‐CypA mAb‐treated mice, respectively. On the basis of established cell type‐specific markers, we resolved 33 transcriptional clusters corresponding to 13 major cell types, including T lymphocyte (T cell), EC, pericyte, B lymphocyte (B cell), natural killer (NK) cell, aerocyte, fibroblast, alveolar type 2 (AT2) cell, macrophage, alveolar type 1 (AT1) cell, monocyte, club cell, and neutrophil (Figure 2A and Figure S3A,B). Because AT2 cells are primary targets for respiratory bacterial adhesion, we focused on their transcriptional changes. Compared with the PBS controls, the eCypA treatment resulted in 1243 up‐ and 1062 downregulated genes in AT2 cells. Cotreatment with the anti‐CypA mAb shifted this to 822 up‐ and 1405 downregulated genes relative to that in response to eCypA alone (Figure 2B and Figure S3C). Importantly, eCypA selectively upregulated key respiratory bacterial adhesion receptors, such as *Itga5*, *Cd46*, *Fn1*, *Ptafr*, and *Muc1*, and this effect was suppressed by the anti‐CypA mAb (Figure 2C). Because integrin α5 and Fn1 are among the most extensively documented adhesion receptors involved in respiratory pathogen attachment, particularly for the two pathogens emphasized in this study (GAS and *S. P*.), we next established a non‐infection model (Figure 2D) to measure the effect of eCypA on integrin α5 and Fn expression. eCypA significantly increased both integrin α5 and Fn at the mRNA (Figure 2E) and protein (Figure 2F) levels, and the anti‐CypA mAb blocked these effects. This effect of eCypA was also confirmed in cell models. Parallel experiments in A549 (Figure 2G,H) and PHTF (Figure S3D,E) cell lines confirmed eCypA‐driven upregulation of bacterial adhesion receptors, which was likewise inhibited by anti‐CypA mAb. Moreover, because IAV also stimulates eCypA secretion, the administration of anti‐CypA mAb suppressed the IAV‐driven increase in integrin α5 and Fn expression in both mouse (Figure S4A,B) and cell (Figure S4C,D) models. These data demonstrate that eCypA promotes the adherence of GAS and *S. P*. by upregulating integrin/ECM adhesion receptors.

**FIGURE 2:**
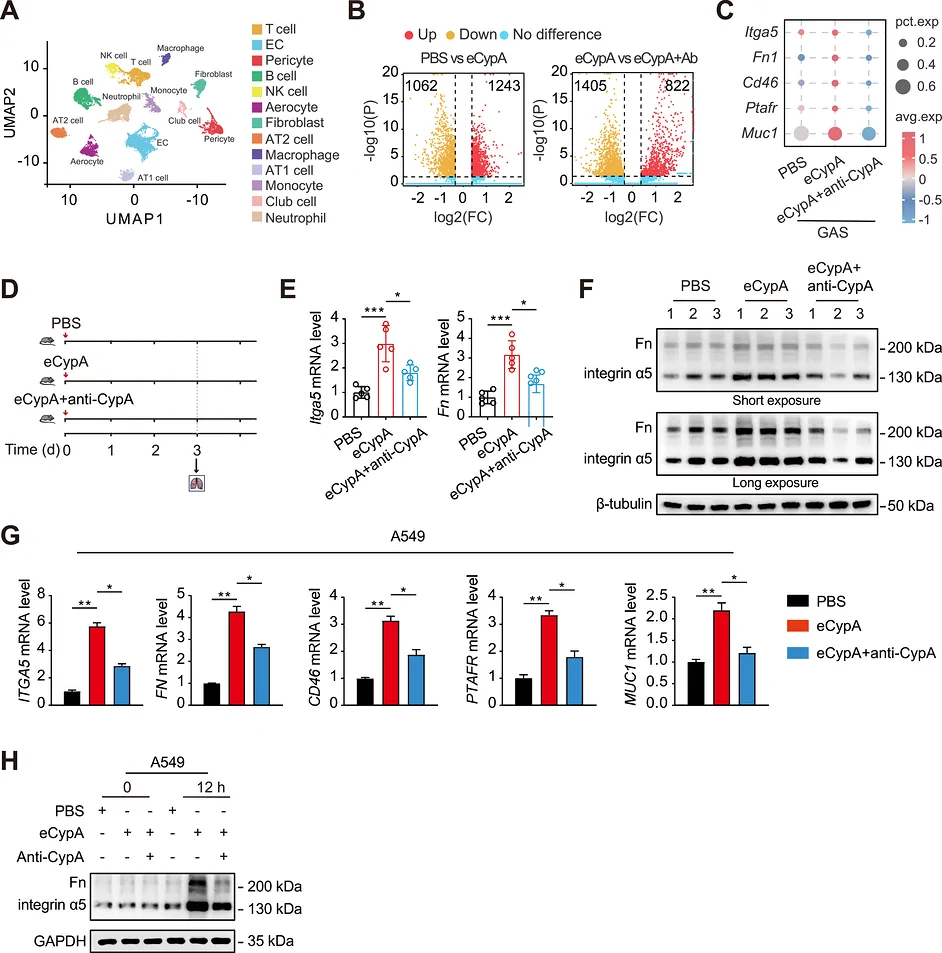
eCypA upregulates the expression of bacterial adhesion receptors. (A) UMAP visualization of pooled snRNA‐seq data from 37,561 cells isolated from lung tissues of mice treated with PBS, eCypA, or eCypA + anti‐CypA mAb, and then infected with GAS. (B) Volcano plots illustrating the DEGs in alveolar type 2 (AT2) cells from PBS‐, eCypA‐, and eCypA + anti‐CypA mAb‐treated mice. (C) Dot plots illustrating the expression of the indicated genes in AT2 cells from PBS‐, eCypA‐, and eCypA + anti‐CypA mAb‐treated mice. (D) C57BL/6 mice were preinjected intraperitoneally with eCypA (40 µg/mouse) or vehicle (PBS) for 2 h, followed by intraperitoneal injection of anti‐CypA mAb (100 µg/mouse) or vehicle (PBS) on Day 0. (E and F) qPCR (E) and immunoblot (F) analyses of integrin α5 and Fn expression in the lungs of C57BL/6 mice (*n* = 5) on Day 3. (G) qPCR analysis of *ITGA5*, *FN*, *CD46*, *PTAFR*, and *MUC1* mRNA levels in A549 cells treated with PBS, eCypA (10 nM), or eCypA + anti‐CypA mAb (10 nM eCypA + 50 nM anti‐CypA mAb, preincubated at 37°C for 1 h) for 12 h. (H) Immunoblot analysis of the indicated proteins in A549 cells as described in G. Data shown in (E) are presented as the means ± SEMs; Data shown in (G) are presented as the means ± SDs; *p < 0.05, **p < 0.01, and ***p < 0.001. One‐way ANOVA followed by Dunnett's test for (E) and (G).

### 2.3 eCypA Promotes Integrin/ECM Adhesion Receptor Expression by Activating TGF‐β Signaling

We next investigated how eCypA drives the upregulation of bacterial adhesion receptors. KEGG pathway analysis of the differentially expressed genes (DEGs) in AT2 cells revealed enrichment in focal adhesion, adherens junction, and actin cytoskeleton regulation pathways (Figure 3A). A Venn analysis comparing genes upregulated by eCypA with those downregulated by anti‐CypA mAb revealed a significant overlap (Figure 3B). KEGG enrichment analysis of these overlapping genes revealed that adhesion and inflammatory pathways, with the TGF‐β signaling cascade, known to regulate integrin and ECM component expression, were prominently enriched (Figure 3C). Parallel Gene Ontology (GO) analysis further suggested multiple routes by which eCypA may engage in TGF‐β signaling (Figure 3D).

**FIGURE 3:**
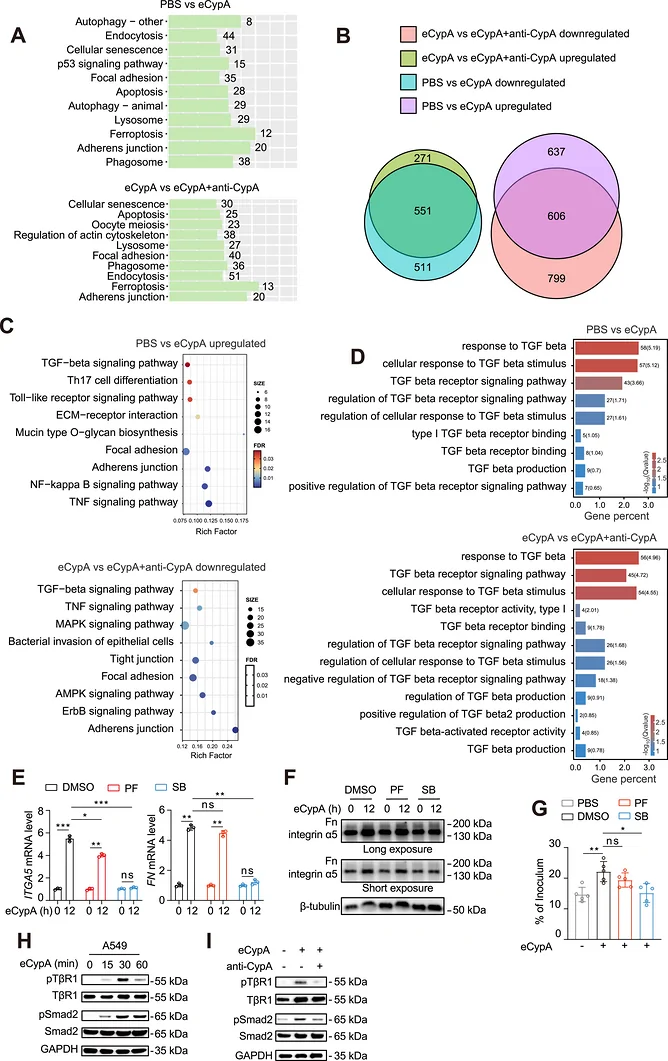
eCypA promotes integrin/ECM adhesion receptor expression by activating TGF‐β signaling. (A) Frequency of enriched KEGG pathways in AT2 cells from the lungs of mice across different treatment groups: PBS versus eCypA and eCypA versus eCypA+anti‑CypA. (B) Venn diagram depicting the overlap in AT2 cells from the lungs of mice across different treatment groups: PBS versus eCypA and eCypA versus eCypA+anti‐CypA mAb. Numbers indicate the count of unique or shared gene sets in each segment (e.g., 271 gene sets are uniquely upregulated in eCypA versus eCypA + anti‐CypA mAb; 551 are shared upregulated sets across both comparisons). (C) Dot plots illustrating KEGG pathways enriched among upregulated genes in AT2 cells from PBS‐ versus eCypA‐treated mice and among downregulated genes from eCypA‐ versus eCypA+anti‑CypA‐treated mice. (D) Bar plots illustrating GO enrichment for DEGs in AT2 cells from the lungs of mice across different treatment groups: PBS versus eCypA and eCypA versus eCypA+anti‑CypA. (E) qPCR analysis of *ITGA5* (left) and *FN* (right) mRNA levels in A549 cells pretreated with DMSO, PF, or SB for 2 h, followed by treatment with eCypA (10 nM) for 12 h. (F) Immunoblot analysis of the indicated proteins in A549 cells as described in E. (G) Adherence assays of GAS in A549 cells treated as described in E and infected with GAS (MOI = 10.0) for 2 h. Data are reported as the percentage of adherent bacteria relative to the input inoculum. (H) Immunoblot analysis of the indicated proteins in A549 cells treated with eCypA (10 nM) for various durations. (I) Immunoblot analysis of the indicated proteins in A549 cells treated with eCypA (10 nM), or eCypA (10 nM) + anti‐CypA mAb (50 nM) for 30 min. Data are presented as the means ± SDs; *p < 0.05, **p < 0.01, ***p < 0.001, and ns = not significant. One‐way ANOVA followed by Dunnett's test for (E) and (G).

The TGF‐β/Smad and FAK signaling pathways control integrin/ECM component expression during respiratory infection [13, 23, 44]. To confirm the involvement of these pathways, we treated cells with the TGF‐β receptor inhibitor SB431542 (SB) or the FAK inhibitor PF573228 (PF) prior to eCypA exposure. Real‐time PCR and western blotting revealed that SB, but not PF, strongly diminished the eCypA‐induced upregulation of integrin α5 and Fn expression (Figure 3E,F) and abolished the increase in GAS adherence (Figure 3G). Furthermore, eCypA significantly increased the phosphorylation of TGF‐β type I receptor (TβR1), a critical step for TGF‐β signaling activation [45, 46, 47], as well as its downstream effector Smad2 (Figure 3H), and these effects were reversed by anti‐CypA mAb (Figure 3I). Moreover, the snRNA‐seq data revealed that eCypA upregulated the expression of both TβR1 and TβR2, but this effect was suppressed by the anti‐CypA mAb (Figure S5A). In A549 cells, eCypA dose‐dependently increased the expression of TβR1 and especially TβR2 at the mRNA (Figure S5B) and protein (Figure S5C) levels. Finally, in CypA‐knockdown 293T cells, exogenous eCypA similarly elevated the expression of TβR1 and TβR2 (Figure S5D). Collectively, these results confirm that eCypA promotes the expression of bacterial adhesion receptors by activating the TGF‐β signaling pathway.

### 2.4 Binding of eCypA to TβR2 Facilitates the Formation of the TGFβ1–TβR2 Complex

The activity of eCypA requires specific cell surface receptors, including CD147, integrin β2, ACE2, and CD146 [37, 39, 40], but whether it requires TGF‐β receptors remains unclear. To address this, we knocked down TβR2, CD147, ACE2, or CD146 in A549 cells and then measured eCypA‐induced integrin α5 and fibronectin expression. Only TβR2 depletion markedly blunted these responses (Figure 4A), indicating that the proadhesive effect of eCypA is specifically dependent on TGF‐β receptor signaling. We therefore examined the interaction of eCypA with TGF‐β receptors. The R55 amino acid residue of CypA is crucial for its PPIase activity, and mutation of R55A results in low PPIase catalytic activity (0.1%) [48]. An immunoprecipitation assay revealed that more TβR1 and TβR2 in A549 cells were pulled down by Fc‐tagged CypA (CypA‐Fc) than by R55A mutant Fc‐tagged CypA (R55‐Fc) (Figure 4B), and that anti‐CypA mAb treatment inhibited the interaction of the CypA protein with TβR1 and TβR2. These results indicate that CypA interacts with TβR1 and TβR2, either directly or indirectly, and that its PPIase activity is critical for this interaction.

**FIGURE 4:**
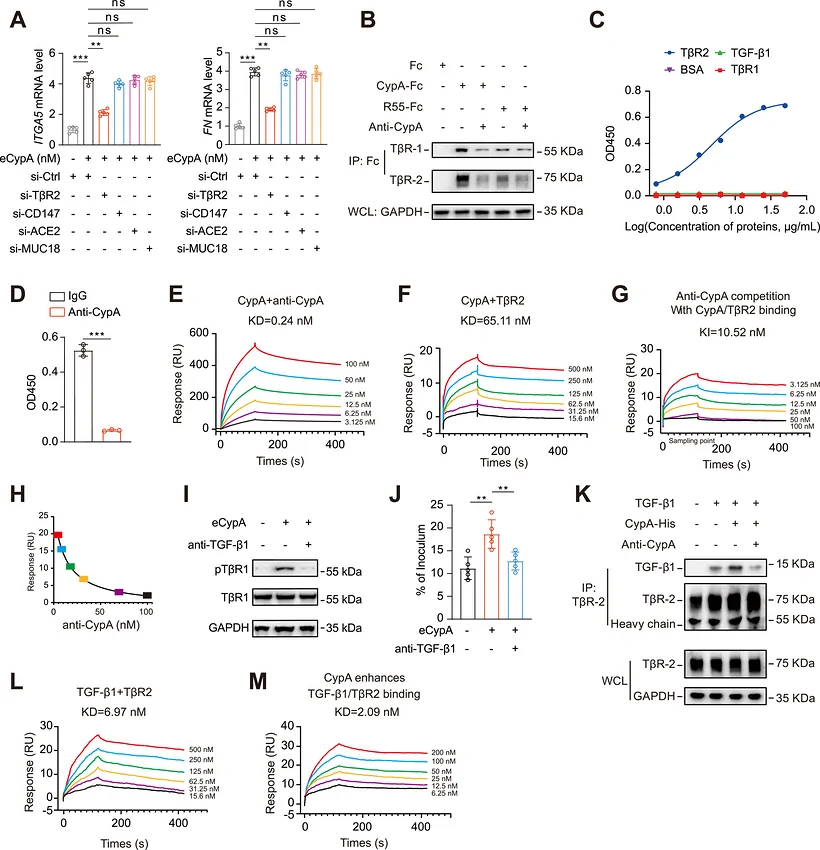
Binding of eCypA to TβR2 facilitates the formation of the TGFβ1‐TGF‐β receptor 2 complex (A) qPCR analysis of *ITGA5* and *FN* mRNA levels in A549 cells transfected with siRNAs targeting TβR2, CD147, ACE2, or MUC18 for 48 h, and then treated with 10 nM eCypA or PBS for 12 h. (B) Immunoblot analysis of the indicated proteins in immunoprecipitated samples and whole‐cell lysates (WCL) of A549 cells treated with Fc, CypA‐Fc (200 ng/mL), R55‐Fc (200 ng/mL), CypA‐Fc + anti‐CypA mAb (1 µg/mL), or R55‐Fc + anti‐CypA mAb for 2 h. (C) Interactions of CypA with TGF‐β1, TβR1, and TβR2 were detected using ELISA. BSA was used as a negative control. (D) The blocking effect of the anti‐CypA mAb on the binding of CypA to TβR2 was detected using ELISA. (E–H) Interaction of CypA with anti‐CypA mAb (E) and TβR2 (F) was detected by SPR. The competitive effect of anti‐CypA mAb on CypA‐TβR2 interaction was detected using competitive SPR assay (G). Linearities of the CypA‐TβR2 interaction were generated from the data of the sampling points shown in (H). (I) Immunoblot analysis of the indicated proteins in A549 cells treated with eCypA (10 nM) or eCypA (10 nM) + TGF‐β pAb (50 nM) for 30 min. (J) Adherence assays of GAS in A549 cells treated with eCypA (10 nM) or eCypA (10 nM) + TGF‐β pAb (50 nM) for 12 h, and then infected with GAS (MOI = 10.0) for 2 h. Data are reported as the percentage of adherent bacteria relative to the input inoculum. (K) Immunoblot analysis of the indicated proteins in immunoprecipitated samples and WCLs of A549 cells treated with TGF‐β1 (5 ng/mL), TGF‐β1 + CypA‐His (10 nM), or TGF‐β1 + CypA‐His + anti‐CypA mAb (50 nM) for 2 h. (L and M) Interactions of TGF‐β1 with TβR2 were detected by SPR in the absence (L) or presence (M) of CypA. Data are presented as the means ± SDs; **p < 0.01, ***p < 0.001 and ns = not significant; unpaired two‐tailed Student's *t* test for (D); one‐way ANOVA followed by Dunnett's test for (A) and (J).

Using AlphaFold3 [49], we predicted the structure of the CypA–TβR1–TβR2 ternary complex (Figure S6A). To experimentally validate this predicted interaction, His‐tagged TGF‐β1 and the extracellular fragment of TβR1 and TβR2 were transiently expressed in HEK293F cells and purified using immobilized metal affinity chromatography (Figure S6B). The ELISA results revealed that CypA bound to TβR2 but not to TGF‐β1 or TβR1 (Figure 4C) and that the anti‐CypA mAb blocked the binding of CypA to TβR2 (Figure 4D). Surface plasmon resonance (SPR) and competitive SPR assays confirmed the binding affinity of CypA for TβR2 and the inhibitory effect of the anti‐CypA mAb on the CypA–TβR2 interaction. CypA showed high binding affinity for the anti‐CypA mAb (*K_D_* = 0.24 nM) (Figure 4E) and bound to TβR2 (*K_D_* = 32.03 nM) in a concentration‐dependent manner (Figure 4F). Furthermore, anti‐CypA mAb blocked the CypA‐TβR2 interaction (*K_i_* = 8.57 nM) in a concentration‐dependent manner (Figure 4G,H). These results indicate that CypA directly binds to TβR2.

We confirmed that the CypA induced increase in GAS adherence is dependent on TGF‐β1. The anti‐TGF‐β1 polyclonal antibody inhibited eCypA‐induced phosphorylation of TβR1 (Figure 4I) and abolished the increase in GAS adherence (Figure 4J) in A549 cells. TGF‐β1 initiates signaling through binding to TβR2 on the cell surface. Therefore, we investigated whether eCypA binding to TβR2 could facilitate the binding of TGFβ1 to TβR2. The immunoblotting results revealed that eCypA significantly increased the binding of TβR2 to TGFβ1, which was attenuated by the anti‐CypA mAb (Figure 4K). In addition, SPR assays were performed to confirm the effect of eCypA on the interaction between TGF‐β1 and the TGF‐β receptor. The results revealed that the binding of TGF‐β1 to TβR2 (Figure 4L) was significantly enhanced by eCypA (Figure 4M). Together, these results indicate that eCypA acts as a coactivator of TβR2 to facilitate the formation of the TGFβ1–TβR2 complex, thereby promoting downstream signal transduction.

### 2.5 Anti‐CypA mAb Exerts Therapeutic Effects Against GAS and *S. P*. Infection

We further examined whether anti‑CypA could inhibit bacterial infection in mice by blocking the ability of eCypA to upregulate adhesion receptors expression and bacterial adherence. Mice were infected with GAS or *S. P*. and then treated with IgG, antibiotics (azithromycin, AZM), anti‐CypA mAb, or a combination of antibiotics and anti‐CypA mAb the next day. On 4 d.p.i., the mouse lungs were collected to detect CFUs and bacterial receptor expression (Figure 5A). Anti‐CypA mAb exhibited an effect similar to that of AZM in terms of the suppression of GAS and *S. P*. adherence. Compared with single treatments, AZM plus anti‐CypA mAb markedly reduced GAS and *S. P*. adherence by 15‐ to 30‐fold (Figure 5B,C). Moreover, AZM and anti‐CypA mAb, particularly the combination of AZM and anti‐CypA mAb, decreased GAS‐induced integrin α5 and Fn expression at both the transcriptional (Figure 5D,E) and protein levels (Figure 5F). These results are consistent with those of the bacterial adherence assay.

**FIGURE 5:**
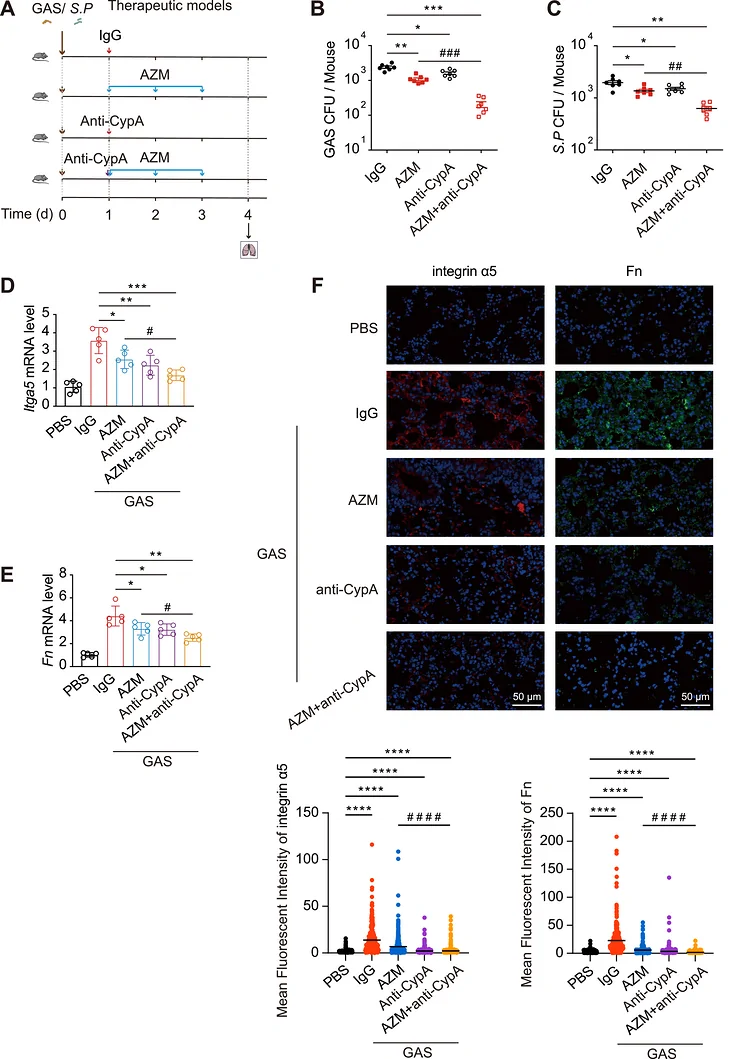
Anti‐CypA mAb suppresses the adherence of GAS and *S. P*. (A) C57BL/6 mice were infected intranasally with GAS (1.0 × 10^7^ CFU), *S. P* (1.0 × 10^7^ CFU), or PBS on Day 0 followed by intraperitoneal injection of IgG, AZM (200 µg/mouse/24 h), anti‐CypA mAb (100 µg/mouse), or AZM (200 µg/mouse/24 h) + anti‐CypA mAb (100 µg/mouse) on Day 1. The same treatment with AZM was administered on Days 2 and 3. (B–C) Adherence of GAS (B) and *S. P* (C) in the lungs of mice (*n* = 7) on Day 4. (D–E) qPCR analysis of *Itga5* (D) and *Fn* (E) mRNA levels in the lungs of mice (*n* = 5) on Day 4. (F) Immunostaining of integrin α5 and Fn in the lungs of mice (*n* = 5) on Day 4 (top; blue: DAPI; red: integrin α5; green: Fn). Scale bar, 50 µm. Quantitative analysis of the mean fluorescence intensity for integrin α5 and Fn per cell (below). Data are presented as the means ± SEMs; *p < 0.05, **p < 0.01, and ***p < 0.001 (one‐way ANOVA followed by Dunnett's test); #*p* < 0.05, ##*p* < 0.01, and ###*p* < 0.001 (unpaired two‐tailed Student's *t* test).

In addition, the effect of anti‐CypA mAb on GAS‐induced lung injury in mice was further investigated. At 4 d.p.i., the production of 36 cytokines in the BALF of GAS‐infected mice was detected using ProcartaPlex Multiplex Immunoassays. Cytokine levels (Figure 6A), particularly those of most proinflammatory cytokines (Figure 6B), were significantly lower in the BALF of mice treated with anti‐CypA mAb or AZM + anti‐CypA mAb than in that of mice treated with IgG. The levels of major proinflammatory cytokines in the BALF were further examined using an enzyme‐linked immunosorbent assay (ELISA). Interleukin (IL)‐1β, IL‐6, tumor necrosis factor (TNF‐α), and CCL2 were reduced in the BALF of mice treated with anti‐CypA mAb or AZM + anti‐CypA mAb (Figure 6C), which was consistent with the results shown in Figure 6B. Next, we investigated the effect of anti‐CypA mAb on the pathogenicity of GAS infection. The lung indices (100× lung/body weight) (Figure 6D) and weight loss (Figure 6E) of anti‐CypA mAb and AZM + anti‐CypA mAb‐treated mice were lower than those of IgG‐treated mice. Lung injury was evaluated in GAS‐infected mice. Hematoxylin and eosin staining indicated that the anti‐CypA mAb and AZM alleviated lung consolidation and resulted in less leukocyte infiltration, hemorrhage, and edema (Figure 6F). Notably, compared with either treatment alone, the combination of AZM and the anti‐CypA mAb enhanced the therapeutic effect against bacterial‐induced lung injury. These results demonstrate that anti‑CypA inhibits pulmonary GAS and *S. P*. infection by blocking eCypA‑mediated upregulation of bacterial adherence.

**FIGURE 6:**
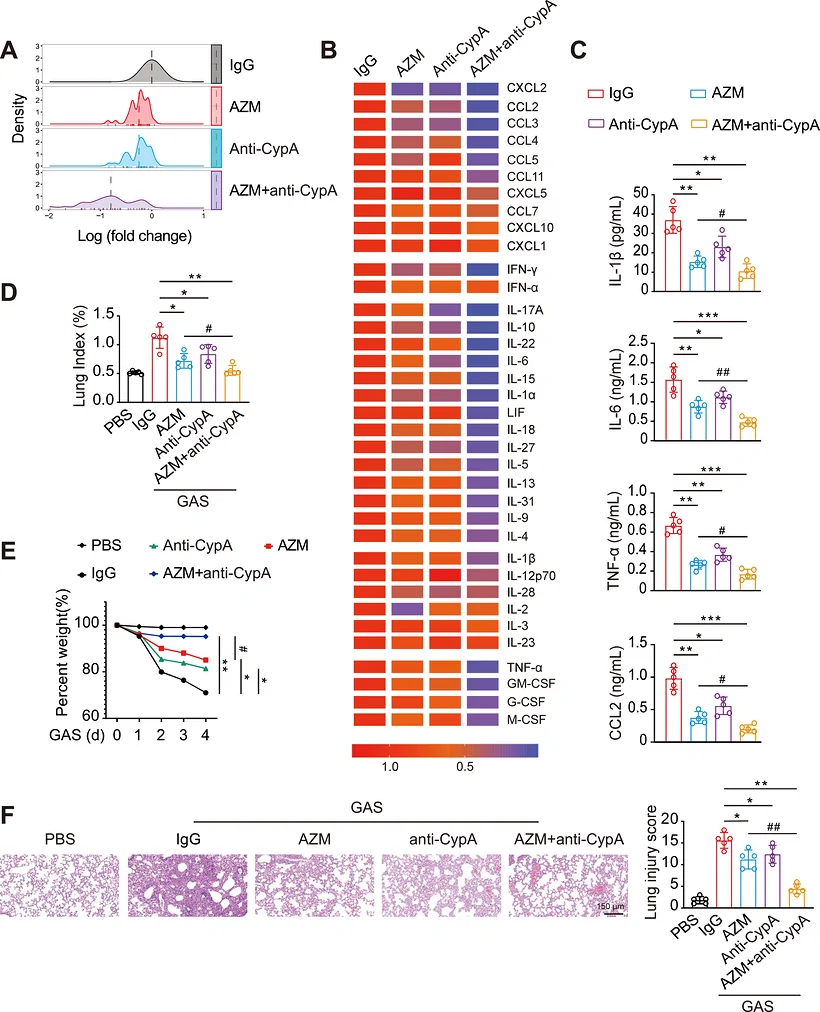
Anti‐CypA mAb alleviates bacterium‐induced cytokine storm. (A–B) ProcartaPlex multiplex immunoassays of cytokines in the BALF of mice (*n* = 5) as described in Figure 5A. Ridge plot of the log_2_ fold‐change density distribution. Dotted lines indicate the mean log_2_ fold‐change (A). Heatmap of cytokine panels altered in the different groups at the indicated time points (B). (C) ELISA of proinflammatory cytokines in the BALF of the mice described in Figure 5A. (D) The lung index (100× lung/body weight) was calculated on Day 4. (E) The body weight of mice on Days 0–4. (F) Hematoxylin and eosin staining of mouse lungs (left). The severity of lung injury was analyzed in a blinded manner (right). Scale bar, 150 µm. Data are presented as the means ± SEMs; *p < 0.05, **p < 0.01, and ***p < 0.001 (one‐way ANOVA followed by Dunnett's test); #*p* < 0.05, and ##*p* < 0.01 (unpaired two‐tailed Student's *t* test).

### 2.6 Anti‐CypA mAb as a Protective Agent Against GAS Infection

We also investigated the prophylactic effects of anti‐CypA mAb on GAS adherence during GAS infection. The mice were pretreated with anti‐CypA mAb or IgG and infected with GAS on the following day. At 4 d.p.i., the mouse lungs were collected to detect CFUs and bacterial receptor expression (Figure 7A). Adherence to GAS (Figure 7B) and expression of its receptors (integrin α5 and Fn) (Figure 7C,D) were significantly lower in the lungs of anti‐CypA mAb‐pretreated mice than in those of IgG‐pretreated mice. In addition, pretreatment with anti‐CypA mAb reduced proinflammatory cytokine expression (Figure 7E) and lung injury (Figure 7F) in GAS‐infected mice. Thus, anti‐CypA mAb may be a useful protective agent against bacterial infections.

**FIGURE 7:**
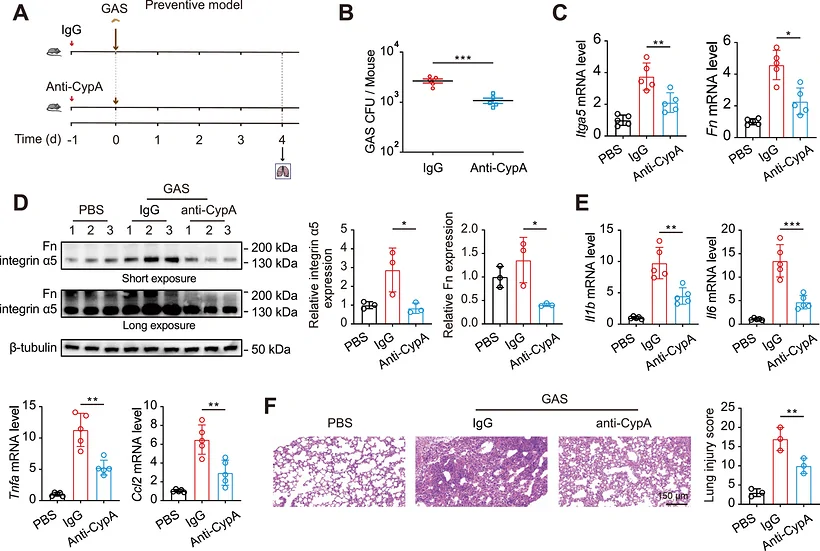
Anti‐CypA mAb as a protective agent against bacterial infection. (A) C57BL/6 mice were preinjected intraperitoneally with IgG or anti‐CypA mAb (100 µg/mouse) and infected intranasally with GAS (1.0 ×10^7^ CFU) on the next day. (B) Adherence of GAS in the mouse lungs (*n* = 5) on Day 4. (C) qPCR analysis of *Itga5* and *Fn1* mRNA levels in mouse lungs (*n* = 5) on Day 4. (D) Immunoblot analysis of integrin α5 and Fn protein levels in mouse lungs (*n* = 3) on Day 4 (left). The relative expression levels of integrin α5 and Fn were quantified (right). (E) ELISA of proinflammatory cytokines in the BALF of mice (*n* = 5). (F) Hematoxylin and eosin staining of mouse lungs (left). The severity of lung injury was analyzed in a blinded manner (right). Scale bar, 150 µm. Data are presented as the means ± SEMs; *p < 0.05, **p < 0.01, and ***p < 0.001, unpaired two‐tailed Student's *t* test.

## 3 Discussion

Antimicrobial resistance is a critical global health concern. Targeting host factors rather than bacteria may largely avoid selective pressure for the evolution of bacterial resistance. In the present study, we revealed that eCypA is a promising HDT target for bacterial infections such as GAS and *S. P*., which require integrins and the ECM as receptors for adherence. IAV or bacterium‐induced eCypA promoted GAS and *S. P*. adherence and then aggravated mouse lung injury by upregulating the expression of adhesion receptors, such as integrin α5 and Fn. Moreover, compared with either treatment alone, the anti‐CypA mAb had an effect similar to that of AZM in suppressing bacterial infection, and the combination of the anti‐CypA mAb with antibiotics resulted in enhanced therapeutic effects. These findings demonstrate that the induction of host cells to secrete eCypA is a previously unknown mechanism through which many bacteria enhance their adherence to host cells. We also provide a promising HDT target for the treatment of bacterial infections.

In this study, we uncover an unexpected extracellular checkpoint that links eCypA to a canonical tissue‐remodeling pathway: eCypA binds to TβR2 and functions as a coactivator, enhancing TβR2–TGF‐β1 engagement and amplifying TGF‐β/Smad signaling, thereby driving adhesion receptor expression. This finding establishes a direct molecular connection between a stress‐inducible secreted host factor and the TGF‐β–dependent control of epithelial adhesiveness, providing a unifying explanation for how diverse bacteria that rely on integrin/ECM receptors can benefit from host responses to viral or bacterial challenge. Because TGF‐β signaling can upregulate the expression of many integrins and ECM components [44], eCypA may represent a broadly acting regulator of bacterial infection for pathogens that depend on integrin/ECM‐mediated adherence. Notably, in addition to integrin α5 and Fn, eCypA upregulated other key bacterial adhesion receptors, including CD46, PAFR, and Mucin1, which are known to facilitate the attachment of respiratory pathogens, including GAS, *S. P*., *S. A*., *Pseudomonas aeruginosa*, and *H. influenzae* [14, 16, 50, 51, 52]. These findings further support the broad role of eCypA in modulating host adhesion machinery to promote colonization by diverse bacterial species. In addition, because sustained activation of TGF‐β/Smad signaling is a major driver of fibrosis progression [53], our data suggest that targeting eCypA may have therapeutic potential in fibrosis‐related diseases and warrants further investigation.

In addition to promoting bacterial adhesion receptor expression, eCypA secreted from cells can bind to integrin β2 and CD147 to activate inflammatory signaling pathways, leading to leukocyte trafficking and proinflammatory cytokine production during viral infection or some chronic inflammatory diseases [35, 37, 54]. These results indicate that eCypA regulates infectious inflammatory diseases via different mechanisms. Therefore, targeting eCypA may not only block bacterial adherence but also inhibit inflammatory responses during bacterial infections.

Secondary bacterial coinfections with IAV and other respiratory viruses are frequently observed and are the leading causes of death in clinical cases [55, 56, 57, 58]. Increased bacterial colonization of the upper respiratory tract is the initial step of coinfection [59]. Our previous study revealed that iCypA interacts with FAK and positively regulates the IAV‐activated FAK signaling pathway, thereby promoting integrin α5 expression and facilitating GAS adherence [23]. In the current study, we found that eCypA regulates bacterial adherence independently of iCypA (Figure S2A,B). Binding of eCypA to TβR2 upregulates the TGF‐β signaling pathway and subsequently promotes the expression of many adhesion receptors, such as integrin α5 and Fn, which are required for the colonization of many respiratory bacteria. These findings suggest that eCypA is a therapeutic target for respiratory virus‐induced bacterial coinfection.

In conclusion, eCypA is an inducible host factor during respiratory bacterial infection and functions as a coactivator of TβR2 to increase TGF‐β/Smad signaling through binding to TβR2, leading to increased bacterial adherence. In addition, anti‐CypA mAb reduced GAS and *S. P*. adherence and bacterium‐induced inflammatory responses and enhanced the therapeutic effects of antibiotics in the treatment of bacterial pneumonia. Notably, the protective effect of anti‐CypA mAb against bacterial infection in mice highlights its promise as a prophylaxis for at‐risk populations, including patients with IAV‐bacterial coinfection and those with autoimmune diseases receiving immunosuppressive therapy. These findings reveal a conserved host mechanism that governs the earliest step of infection and attachment and highlight a promising HDT for bacterial infection.

Several limitations should be noted. First, although our current results confirm the selectivity of the anti‐CypA mAb in specific bacterial contexts and suggest therapeutic potential, broader validation is required to conclusively establish a conserved regulatory role for eCypA. Second, while our data suggest that eCypA functions predominantly through TβR2‐mediated TGF‐β signaling, we cannot exclude the involvement of other pathways that intersect with the TGF‐β axis. Third, despite demonstrating direct CypA–TβR2 binding, the precise binding interface and the key residues of TβR2 involved remain to be defined. Addressing these limitations in future studies will not only refine our mechanistic model but also facilitate the rational design of inhibitors targeting the CypA–TβR2 interaction and further support eCypA as a promising candidate for host‐directed antibacterial therapy.

## 4 Materials and Methods

### 4.1 Mice

Female C57BL/6 mice (6–8 weeks old) were purchased from the Vital River Laboratory Animal Center (Beijing, China) and maintained under specific pathogen‐free conditions at the Institute of Microbiology. All animals were housed in a barrier facility under standard conditions, with a 12 h light/12 h dark cycle, an ambient temperature of 22 ± 2°C, and a relative humidity of 40%–70%. Mice were randomized to groups (*n* = 5–7 per group; 10–28 per experiment; total *n* = 97 across six experiments) via a computer‐generated sequence. Sample size was based on pilot data and the minimum needed for valid analysis per the 3Rs. Allocation was performed by an independent investigator; treatment/measurement orders were balanced. Due to the intervention, experimenters were not blinded to group assignment, while outcome assessment and analysis were conducted by blinded investigators. No inclusion or exclusion criteria were specified. All animal experiments were approved by the Research Ethics Committee of the Institute of Microbiology (APIMCAS2023052) and complied with the Beijing Laboratory Animal Welfare and Ethical Guidelines of the Beijing Administration Committee of Laboratory Animals.

### 4.2 Cell Lines

A549 cells (ATCC CCL185), Madin‐Darby canine kidney cells (MDCKs, ATCC CCL‐34), and HEK293T cells (human embryonic kidney cells, ATCC CR‐3216) were purchased from the American Type Culture Collection (ATCC, Manassas, VA, USA). PHTF (CTCC‐075‐HUM) were purchased from Meisen Chinese Tissue Culture Collection (MeisenCTCC, Zhejiang, China). Short hairpin RNA‐based knockdown of CypA in A549 and 293T cells has been previously described [60]. MDCKs were used for IAV titration. All the cell lines were tested for mycoplasma contamination using a MycoAlert mycoplasma detection kit (Lonza, Basel, Switzerland) and maintained in Dulbecco's modified Eagle's medium (DMEM, Gibco, Grand Island, NY, USA) supplemented with 10% (v/v) fetal bovine serum (Gibco) at 37°C under a humidified atmosphere containing 5% CO_2_.

### 4.3 Bacteria and Virus Strains

The GAS strain 90–226 (M1 strain), *K. P*. strain 10617, and *S. P*. strain T19F were kindly provided by Professor Beinan Wang (Chinese Academy of Sciences, China). GAS and *S. P*. were maintained on blood agar with 15% (v/v) sheep blood and grown in THB‐Neo medium at 37°C. *K. P*. was grown in tryptic soy broth (Difco, Detroit, MI, USA) for 16 h at 37°C. For CFU counting assays, the bacteria were grown overnight. Influenza A/WSN/33 (H1N1) was obtained from cDNA [61], cultured in the allantoic cavities of 9‐day‐old specific pathogen‐free embryonic eggs, and incubated for 48–72 h at 37°C; the allantoic fluid was subsequently harvested, centrifuged, and stored at −80°C. IAV titration was performed as previously described [23]. Briefly, monolayers of Madin‐Darby canine kidney cells were washed with PBS and inoculated with 10‐fold serially diluted virus with five replicates of each dilution for 2 h. The cells were subsequently washed with PBS and overlaid with phenol red‐free DMEM containing 0.6% low‐melting‐point agarose and 1 mg/mL l‐(tosylamido‐2‐phenyl) ethyl chloromethyl ketone‐treated trypsin (TPCK). After incubation for 48–72 h at 37°C with 5% CO_2_, the IAV titers were determined on the basis of the number of visible plaques.

### 4.4 Antibodies and Proteins

Rabbit polyclonal antibodies against β‐tubulin (Cat# ab6046) were purchased from Abcam. Rabbit polyclonal antibody against integrin α5 (Cat# PA5‐79529) was purchased from Thermo Fisher. A mouse monoclonal antibody against fibronectin (A‐11) (Cat#s c‐271098) was purchased from Santa Cruz Biotechnology. Rabbit monoclonal antibodies against TGF‐beta receptor I (F6L3I) (Cat# 49728), TGF‐beta receptor II (E5M6F) (Cat# 41896), TGF‐beta (56E4) (Cat# 3709), SMAD2 (D43B4) (Cat# 5339), Phospho‐SMAD2 (Ser465/467) (138D4) (Cat# 3108), GAPDH (D4C6R) (Cat# 97166), Flag (D6W5B) (Cat# 14793) were purchased from Cell Signaling Technology. A rabbit polyclonal antibody against phospho‐TGF‐beta receptor I (Ser165) (Cat# 14793) was purchased from CUSABIO. HRP‐conjugated anti‐mouse secondary antibody (Cat# A0216) and HRP‐conjugated anti‐rabbit secondary antibody (Cat# A0208) were obtained from Beyotime. Alexa Fluor 594 goat anti‐rabbit IgG (Cat# 111‐585‐003) and Alexa Fluor 488 goat anti‐mouse IgG (Cat# 115‐545‐003) were purchased from the Jackson Laboratory.

Anti‐CypA mouse mAbs, His‐tagged CypA, Fc‐tagged CypA, and Fc‐tagged CypA‐R55 were obtained as previously described [37]. Mouse IgG (MPC‐11) (Cat#A2145) was purchased from Selleck Chemicals. His‐tagged TGF‐β1, TβR1 (extracellular fragment), and TβR2 (extracellular fragment) were transiently expressed in HEK293F cells and purified using immobilized metal affinity chromatography.

### 4.5 Serum From Patients With Bacterial Infections

This study was conducted in accordance with the principles of the Declaration of Helsinki. Serum was obtained retrospectively from outpatients with GAS or *S. P*. infection (aged 25–50 years, *n* = 12) and from age‐matched healthy donors (*n* = 10) at Nanjing Drum Tower Hospital, The Affiliated Hospital of Nanjing University Medical School. This study was approved by the ethics committees of Nanjing Drum Tower Hospital, The Affiliated Hospital of Nanjing University Medical School (2021‐161‐02).

### 4.6 In Vitro Bacterial Adhesion Assay

A549 cells or PHTF were cultured in 24‐well plates to 90% confluence, washed with PBS, and infected with GAS, *K. P*. or *S. P*. at a multiplicity of infection (MOI) of 10 or 2 in 100 µL of DMEM containing 4% bovine serum albumin. After incubation for 6 h at 37°C with 5% CO_2_, the cells were washed with PBS to remove unbound bacteria, detached using 0.1 mL of trypsin‐ethylenediaminetetraacetic acid (EDTA) solution, and lysed with 0.4 mL of 0.025% Triton X‐100. The lysates were diluted with PBS, and the numbers of CFUs of GAS, *K. P*., and *S. P*. were quantified.

### 4.7 Bacterial Adhesion Assay In Vivo

C57BL/6 mice infected with GAS (1.0 × 10^7^ CFUs), *S. P*. (1.0 × 10^7^ CFU), or *K. P*. (1.0 × 10^7^ CFU) were killed, and the lungs were homogenized in 5 mL of PBS. The homogenate was centrifuged at 500 × *g* for 5 min, the supernatant was discarded, and the pellet was resuspended in 1 mL of 0.025% Triton X‐100. After serial dilution with PBS, GAS, *S. P*., and *K. P*. were quantified as CFUs.

### 4.8 IAV and GAS Coinfection In Vitro

A549 cells were infected with IAV (MOI = 1) in serum‐free DMEM supplemented with 0.5 mg/mL TPCK for 2 h. The medium was replaced with DMEM supplemented with 2% fetal bovine serum, followed by treatment with or without 50 nM anti‐CypA mAb for 12 h and then infected with GAS (MOI = 10).

### 4.9 IAV and GAS Coinfection In Vivo

C57BL/6 mice were anesthetized and inoculated intranasally with IAV (3000 plaque‐forming units) in 30 µL of PBS. After three days, the mice in the coinfection group were anesthetized and inoculated intranasally with GAS (1.0 × 10^7^ CFU).

### 4.10 RNA Isolation and qPCR Analysis

RNA was isolated from A549 cells, PHTF, and mouse lung tissue homogenates using TRIzol reagent (Invitrogen, Carlsbad, CA, USA) in accordance with the manufacturer's protocol. For reverse transcription, 2 µg of total RNA was used as the template, with oligo (dT) primers and M‐MLV reverse transcriptase (Promega, Madison, WI, USA). cDNA was amplified by real‐time PCR using SYBR Premix Ex Taq (TaKaRa Bio, Shiga, Japan). Gene‐specific primer sequences are provided in Table S1. Amplification was performed on an ABI 7500 Real‐Time PCR System (Applied Biosystems, Foster City, CA, USA), and relative transcript abundance was calculated using the 2^−^ *^ΔΔCt^* method.

### 4.11 Western Blotting

A549 cells, PHTF, and mouse lung tissues were lysed in buffer supplemented with 150 mM NaCl, 20 mM HEPES, 1 mM EDTA, 1% Triton X‐100, 10% glycerol, and a protease/phosphatase inhibitor mixture. Following protein extraction, total protein concentrations were determined using a BCA assay kit (Thermo Fisher Scientific, Waltham, MA, USA). Protein samples were subsequently analyzed by western blot according to a previously established protocol [62]. Target protein expression was normalized to that of GAPDH or β‐tubulin.

### 4.12 Gene Silencing

The expression of *TGFBR2*, *BSG*, *ACE2*, and *MCAM* was silenced using target‐specific siRNAs. Specifically, siRNAs targeting *BSG* (sequence: GAGAGTACTCCTGC GTCTT) and *MCAM* (sequence: UGAUAUCGCUGCUGAGUGATT) were synthesized by Sangon Biotech, with CUUCAGCCUCAGCUUGCCGTT used as the negative control. Moreover, siRNAs targeting *TGFBR2* and *ACE2* were purchased from Santa Cruz Biotechnology. All siRNAs were transfected into cells using Lipofectamine RNAiMAX (Thermo Fisher Scientific), and the cells were harvested for subsequent analysis at 48 h post‐transfection.

### 4.13 Direct Binding Assay (ELISA)

Direct binding assays of CypA with TGF‐β1, TβR1, and TβR2 were performed as previously described [36]. Briefly, purified His‐tagged TGF‐β1, TβR1, and TβR2 (1 µg/mL) was added to on 96‐well plates. After treatment with blocking solution, different concentrations of CypA‐Fc were added to the plates. After removal of unbound material by washing, the retained CypA‐Fc was detected with an HRP‐labeled anti‐human heavy‐chain antibody. The reaction signal was quantified by measuring absorbance at 450 nm using a microplate reader. Bovine serum albumin was used as a negative control. For the CypA‐TβR2 interaction blocking assay, purified CypA‐Fc (1 µg/mL) was added to 96‐well plates. After blocking for 1 h with 0.4% bovine serum albumin in PBS containing Tween 20, anti‐CypA mAb (2.5 µg/mL), or IgG1 was added to the plates and incubated for 2 h at room temperature. The plates were subsequently washed and incubated with TβR2‐His for 2 h at room temperature. The plates were then washed three times and incubated with horseradish peroxidase‐conjugated anti‐His (rabbit) antibody for 1 h at room temperature. The plates were incubated with anti‐rabbit secondary antibodies for 1 h. After being washed, the plates were incubated with 3,3′,5,5′, ‐tetramethylbenzidine and the optical density was measured at 450 nm.

### 4.14 Surface Plasmon Resonance

SPR, competitive SPR, and enhanced SPR analyses were performed on a Biacore T200 (Cytiva, Marlborough, MA, USA) as previously described [37]. Briefly, CypA‐Fc was immobilized on the surface of a CM5 sensor chip to detect the binding of CypA with anti‐CypA mAb or TβR2. His‐tagged TβR2 was immobilized on the surface of a CM5 sensor chip to detect the binding of TGFβ1 with TβR2. For the competitive SPR assay, a mixture of CypA (constant concentration, 100 nM) and anti‐CypA mAb (increasing concentration in a gradient) was used as the analyte. For enhanced SPR, a mixture of CypA (constant concentration, 200 nM) and TGF‐β1 (increased concentration in a gradient) was used as the analyte. Binding affinities were detected using kinetic analysis, concentration series, and direct binding modes. The binding and dissociation times were 120 and 300 s, respectively. The results were analyzed using Biacore insight evaluation software 3.2 to determine the affinity or inhibition constants. A steady‐state affinity model was used for fitting analysis.

### 4.15 ProcartaPlex Multiplex Immunoassays and ELISA

For BALF collection, mouse lungs were gently flushed with 1 mL PBS, and the lavage samples were subsequently centrifuged to remove cellular components. The recovered supernatants were used for eCypA and inflammatory mediator measurements. eCypA concentrations in BALF, serum, and cell culture supernatants were quantified by ELISA (CSB‐E09920h, CSB‐E09282m, CUSABIO). BALF cytokines and chemokines were analyzed with a mouse ProcartaPlex multiplex immunoassay kit (EPX360‐26092‐901; Laizee Biotech, Shanghai, China), while the indicated cytokines were additionally quantified using ELISA kits from Multi Sciences (Hangzhou, China).

### 4.16 Histological Analysis

Fixed lung tissues were embedded in paraffin, cut into sections, and subjected to hematoxylin‐eosin staining and immunofluorescence histochemistry. The severity of lung injury was scored in a blinded manner as previously described [23]. Anti‐mouse integrin α5 (Santa Cruz Biotechnology, Dallas, TX, USA), goat anti‐mouse Alexa Fluor Plus 594‐conjugated (red) (Thermo Fisher Scientific) secondary antibodies, anti‐mouse Fn (Santa Cruz Biotechnology), and goat anti‐mouse Alexa Fluor Plus 488‐conjugated (green) (Thermo Fisher Scientific) secondary antibodies were used to detect the expression of integrin α5 and Fn in the lungs of mice.

### 4.17 Single‐Nucleus RNA Sequencing Assays and Data Analysis

Nucleus isolation and snRNA‐seq: Mice were anesthetized with isoflurane, and lungs were collected from each group (pooled from 5 mice per group). Fresh tissues were immediately snap‐frozen in liquid nitrogen and stored at −80°C. Subsequent nuclear isolation and sequencing were performed following the protocol provided by Genedenovo Biotechnology Co. Ltd. (Guangzhou, China) [63].

Data quality control and gene expression quantification: Raw BCL files were converted to FASTQ files, aligned, and quantified using 10× Genomics Cell Ranger software (v3.1.0). Briefly, ineligible reads were filtered out, followed by mapping to the reference genome Ensembl_release111. Only reads uniquely mapped to the transcriptome with at least 50% overlap with an exon were included for UMI counting. Prior to quantification, UMI sequences were corrected for sequencing errors, and valid barcodes were identified using the EmptyDrops method [64]. Cell‐by‐gene matrices were generated via UMI counting and cell barcode calling.

Cell clustering: Cell‐by‐gene matrices of individual samples were imported into Seurat (v3.1.1) [65] for downstream analysis. Cells were filtered out if they had ≥ 19,000 UMIs, ≥ 10% mitochondrial gene content, < 300 detected genes, or > 4500 detected genes. Doublets were removed using the DoubletFinder tool (v2.0.3) [66], which generates artificial doublets, calculates the proportion of artificial k‐nearest neighbors (pANN) for each cell on the basis of PC distance, and ranks cells by the expected doublet number. After filtering, gene expression was normalized using the “log normalize” method (normalization by total expression, multiplication by a scale factor of 10,000, and log‐transformation). Batch effects and behavioral condition‐related biases were minimized using Harmony [67]. Following data integration, principal component analysis (PCA) was performed, and the top 50 principal components were retained for downstream clustering and visualization. Euclidean distances between cells were calculated on the basis of the identified principal components, and cells were embedded into a shared nearest neighbor (SNN) graph. The SNN graph was partitioned into highly interconnected subpopulations using the Jaccard distance between locally proximal cells, and clusters were generated via Louvain's algorithm with a resolution parameter of 0.5. Data were visualized using UMAP (uniform manifold approximation and projection), and cell clusters were annotated mainly on the basis of the expression of canonical marker genes. Specifically, T lymphocytes were identified by *Cd3d*, ECs by *Pecam1*, pericytes by *Cspg4*, B lymphocytes by *Cd19*, NK cells by *Gzma*, aerocytes by *Car4* and *Pecam 1*, fibroblasts by *Pdgfra*, macrophages by *Cd68*, AT2 by *Sftpc*, AT1 by *Hopx*, monocytes by *Ccr2*, club cells by *Ccdc153*, and neutrophils by *S100a8*. The other marker genes of different cell types were described previously [68].

DEG analysis: We used the Wilcoxon rank sum test [69] in Seurat (v3.1.0) to detect the fold change of expression. The MAST package (v1.28.0) was used to assess the significance of differential expression. We identified DEGs according to the following criteria: (1) *p* < 0.05; (2) |log2FC| ≥ 0.36; and (3) the percentage of cells in which the gene was detected in a specific cluster > 10%.

### 4.18 Statistical Analysis

All the statistical analyses were performed using GraphPad Prism 9 (GraphPad, Inc., La Jolla, CA, USA) software and Microsoft Excel (2019). Comparisons were performed as described in the legend. *p* < 0.05 was considered to indicate significant results.

## Author Contributions

L.S., W.L., Z.Z., and Y.C.: supervision; conceptualization; formal analysis; review and editing. X.B. and H.L.: methodology; investigation; formal analysis; original draft preparation. Y.Z. and P.J.: methodology; investigation; formal analysis; review and editing. X.J., H.P., Z.F., and H.Z.: methodology; investigation. R.L.: software; formal analysis. Y.C.: resources. B.W., Y.B., X.C., and D.C.: formal analysis; review and editing. All authors have read and approved the final manuscript.

## Funding

This work was supported by the National Major Science and Technology Project for the Prevention and Control of Emerging and Major Infectious Diseases (2025ZD01903600), National Natural Science Foundation of China (32300145 and 32372986), National Key Research and Development Program of China (2023YFC2308600), Science Fund for Distinguished Young Scholars of Jiangsu Province (BK20250016), and Nanjing Drum Tower Hospital Outstanding Youth Cultivation Fund (2024‐JCY‐YP‐01).

## Ethics Statement

Serum samples were collected in accordance with the Declaration of Helsinki, written informed consent was obtained from all participants, and approval for this study was obtained from the Ethics Committee of Nanjing Drum Tower Hospital, The Affiliated Hospital of Nanjing University Medical School (approval number 2021‐161‐02). All animal experiments were approved by the Research Ethics Committee of the Institute of Microbiology (APIMCAS2023052).

## Conflicts of Interest

W.L., L.S., X.B., and H.Z. have a pending patent related to the anti‐CypA monoclonal antibody described in this work. The applicant is Shenzhen Bay Laboratory. This application claims the benefit of the Chinese Patent Provisional Application No. 2025117736167, filed November 28, 2025.

## Supporting information



Supporting information: mco271063‐sup‐0001‐SuppMat.docx

## Data Availability

The main data supporting the findings of this study are available in the article and its Supporting Information files. Single‐cell RNA‐seq data have been deposited in the Genome Sequence Archive (GSA) with accession number CRA039122.
