# Supplementary material for: Blocking Extracellular Cyclophilin A‐Mediated Adhesion Protects Against Bacterial Infection
Source: MedComm (2020). 2026 Oct 13;7(10):e71063. doi: 10.1002/mco2.71063 (PMC13659286; doi:10.1002/mco2.71063)
Supplement: Supplementary file 1 [file MCO2-7-e71063-s001.docx]

**Blocking extracellular cyclophilin A-mediated adhesion protects against bacterial infection**

Xiaoyuan Bai^1,2#^, Heqiao Li^3#^, Pengtao Jiao^1,4#^, Yuna Zhao^1#^, Xiaoxiao Jia^1,5^, Han Zhong Pei^3^, Zeqiu Fan^1,3^, Runshan Lin^6^, He Zhang^6^, Beinan Wang^1^, Xinhai Chen^3^, Yuhai Bi^1,7^, De Chang^8^, Zhongfa Zhang^9*^, Yuxin Chen^10*^, Wenjun Liu^1,6*^, Lei Sun^1,7,9*^

^1^Key Laboratory of Pathogenic Microbiology and Immunology, Institute of Microbiology, Chinese Academy of Sciences, Beijing 100101, China.

^2^Beijing Institute of Heart, Lung and Blood Vessel Diseases, Beijing Anzhen Hospital, Capital Medical University, Beijing 100029, China.

^3^Institute of Infectious Diseases, Shenzhen Bay Laboratory, Shenzhen 518107, Guangdong, China.

^4^Institute of Animal Science, Chinese Academy of Agricultural Sciences, Beijing 100193, China

^5^National Institute of Pathogen Biology, Chinese Academy of Medical Sciences & Peking Union Medical College, Beijing 102629, China

^6^Shenzhen Medical Academy of Research and Translation (SMART), Shenzhen 518107, Guangdong, China.

^7^Medical School, University of Chinese Academy of Sciences, Beijing 100049, China.

^8^Department of Pulmonary and Critical Care Medicine at The Seventh Medical Center, College of Pulmonary and Critical Care Medicine of The Eighth Medical Center, Chinese PLA General Hospital, Beijing, China

^9^Public Health Clinical Center Affiliated to Shandong University, Shandong Public Health Clinical Center, Jinan 250100, Shandong, China.

^10^Department of Laboratory Medicine, Nanjing Drum Tower Hospital, The Affiliated Hospital of Medical School, Nanjing University, Nanjing 210008, Jiangsu, China.

^#^ These authors contributed equally.

*Correspondence: Zhongfa Zhang, zzf235@163.com; Yuxin Chen, yuxin_chen2015@163.com; Wenjun Liu, liuwenjun@smart.org.cn; Lei Sun, sunlei362@im.ac.cn

**Supplementary Materials**

**Figure S1. Bacterial respiratory infection induces eCypA secretion. Related to Figure 1**

GAS (1.0 × 10^7^ CFUs), *S.P* (1.0 × 10^7^ CFU), or *K.P* (1.0 × 10^7^ CFU) were killed, and the lungs were homogenized in (A) ELISA analysis of eCypA in serum obtained from outpatients with GAS or *S.P* infection (aged 25-50 years, n = 12) and age-matched healthy controls (n = 10). (B) C57BL/6 mice were infected intranasally with GAS (1.0 × 10^7^ CFUs), *S.P* (1.0 × 10^7^ CFU), or *K.P* (1.0 × 10^7^ CFU) on day 0. Serum and BALF were collected from mice (n = 5) at the indicated time points. (C and D) ELISA of eCypA in BALF (C) and serum (D) from mice infected with GAS. (E and F) ELISA of eCypA in BALF (E) and serum (F) from mice infected with *S.P*. (G and H) ELISA of eCypA in BALF (G) and serum (H) from mice infected with *K.P*. (I) ELISA of eCypA in the supernatants of A549 cells and PHTFs infected with GAS (MOI = 2), *S.P* (MOI = 2) or *K.P* (MOI = 2) for 24 h. Data are presented as the means ± SEMs; **p* < 0.05, ***p* < 0.01, and ****p* < 0.001, one-way ANOVA followed by Dunnett’s test (day 0 as a control for multiple comparisons).

**Figure S2. eCypA promotes the adherence of GAS and *S.P* to PHTF and to CypA-knockdown A549 cells. Related to Figure 1**

(A-C) Adherence assays of GAS (A), *S.P* (B), and *K.P* (C) in PHTF cells treated with PBS, eCypA (10 nM) or eCypA + anti-CypA mAb (10 nM eCypA + 50 nM anti-CypA mAb, preincubated at 37 ℃ for 1 h) for 12 h, followed by GAS, *S.P*, or *K.P* (MOI = 10) infection for 2 h. Data are reported as the percentage of adherent bacteria relative to the input inoculum. (D-E) Adherence assays of GAS (D) and *S.P* (E) in A549/CypA- cells treated with PBS or eCypA (10 nM) for 24 h, followed by infection with GAS or *S.P* (MOI = 10) for 2 h. Data are reported as the percentage of adherent bacteria relative to the input inoculum. Data are presented as the means ± SDs; **p* < 0.05 and ns = not significant; unpaired two-tailed Student’s *t* test for (D) and (E); one-way ANOVA followed by Dunnett’s test for (A-C).

**Figure S3 eCypA upregulates the expression of bacterial adhesion receptors Related to Figure 2**

(A) Dot plot showing marker gene expression across various cell subtypes in the lung tissues of mice. (B) Bar plots showing the proportion of each cell type in the mice treated with PBS, eCypA or eCypA + anti-CypA mAb. (C) Bar plots illustrating the DEGs in AT2 cells from PBS-, eCypA-, and eCypA + anti-CypA mAb-treated mice. (D) qPCR analysis of *ITGA5*, *FN*, *CD46*, *PTAFR*, and *MUC1* mRNA levels in PHTF treated with PBS, eCypA (10 nM), or eCypA + anti-CypA mAb (10 nM eCypA + 50 nM anti-CypA mAb, preincubated at 37 ℃ for 1 h) for 12 h. (E) Immunoblot analysis of the indicated proteins in PHTF as described in D. Data are presented as the means ± SDs; **p* < 0.05 and ***p* < 0.01, one-way ANOVA followed by Dunnett’s test.

**Figure S4 Anti-CypA mAb inhibits bacterial adhesion receptor expression during IAV infection. Related to Figure 2**

(A) qPCR analysis of *Itga5* (left) and *Fn* (right) mRNA levels in the lungs of C57BL/6 mice as described in Figure 1A. (B) Immunoblot analysis of integrin α5 and Fn protein levels in the lungs of C57BL/6 mice as described in Figure 1A. (C) qPCR analysis of *ITGA5* (left) and *FN* (right) mRNA levels in A549 cells infected with PBS or IAV (MOI = 1) followed by treatment with IgG or anti-CypA mAb (50 nM) for 24 h. (D) Immunoblot analysis of the indicated proteins in A549 cells as described in C. Data shown in (A) are presented as the means ± SEMS; Data shown in (C) are presented as the means ± SDS; **p* < 0.05, ***p* < 0.01, and ****p* < 0.001. One-way ANOVA followed by Dunnett’s test.

**Figure S5 eCypA promotes TGF-β receptor expression. Related to Figure 3**

(A) Dot plots illustrating the expression of *Tgfbr1* and *Tgfbr2* in AT2 cells from PBS-, eCypA-, and eCypA + anti-CypA mAb-treated mice. (B) qPCR analysis of *TGFBR1* (left) and *TGFBR2* (right) mRNA levels in A549 cells treated with increasing concentrations of eCypA (0, 0.5, 1, 5, 10, or 20 nM) for 12 h. (C) Immunoblot analysis of the indicated proteins in A549/CypA- cells as described in B (left). The relative expression levels of TβR1 and TβR2 were quantified (right). (D) Immunoblot analysis of the indicated proteins in 293T/CypA- cells transfected with Flag-TβR1 and Flag-TβR2 for 24 h, followed by treatment with increasing concentrations of eCypA (0, 0.5, 1, 5, 10, or 20 nM) for 12 h. Data are presented as the means ± SDs; **p* < 0.05, ***p* < 0.01, ****p* < 0.001 and ns = not significant; one-way ANOVA followed by Dunnett’s test.

**Figure S6 eCypA binds to TβR2. Related to Figure 4**

(A) Molecular docking of the CypA-TβR1-TβR2 complex (containing two CypA molecules and a TβR1/TβR2 heterotetramer with two TβR1 and two TβR2 subunits). (B) Expression of His-tagged TGF-β1, and the extracellular fragments of His-tagged TβR1 and TβR2 in the supernatant of HEK293 cells was analyzed using sodium dodecyl sulfate-polyacrylamide gel electrophoresis.

**Table S1. Primers used in this study**

| **Genes** | **Forward primers** | **Reverse primers** |
| --- | --- | --- |
| *ITGA5* | TGCAGTGTGAGGCTGTGTACA | GTGGCCACCTGACGCTCT |
| *FN* | CAGGATCACTTACGGAGAAACAG | GCCAGTGACAGCATACACAGTG |
| *PTAFR* | TACCAACACAGTGCCCGACAGT | ATGAGGAAGACCAGGAAGAAGC |
| *MUC1* | TGCTCCTACAAGTTGGCAGAAGTGG | AACCCGAGACGCCTACCATCCTATG |
| *CD46* | CCTACTTACAAGCCTCCAGTC | GGCATATTCAGCTCCACCATC |
| *TGFBR1* | TGAAGAAATGAGAAAAGTTGTT | CCTGTTGACTGAGTTGCGATAA |
| *TGFBR2* | ATTATGAAGGAAAAAAAAAAGC | GACTAGCAACAAGTCAGGATTG |
| *GAPDH* | TTGTCTCCTGCGACTTCAACAG | GGTCTGGGATGGAAATTGTGAG |
| *Itga5* | GCAGCTTCCCCAAAAGAAACTT | ACCTAGGAGCAGGAGGCCAAAA |
| *Fn1* | ACATGGCTTTAGGCGGACAA | GGCCCGGAACATGAGGATAG |
| *Tnfa* | CATCTTCTCAAAATTCGAGTGACAA | TGGGAGTAGACAAGGTACAACCC |
| *Il1b* | GTGGCTGTGGAGAAGCTGTG | GAAGGTCCACGGGAAAGACAC |
| *Il6* | CCAGAAACCGCTATGAAGTTCC | TTGTCACCAGCATCAGTCCC |
| *Ccl2* | CAGCCAGATGCAATCAATGCC | TGGAATCCTGAACCCACTTCT |
| *Cxcl10* | CCAAGTGCTGCCGTCATTTTCT | TTCCCTATGGCCCTCATTCTCA |
| *Gapdh* | TGTGGATGGCCCCTCTGGAA | TGACCTTGCCCACAGCCTTG |
